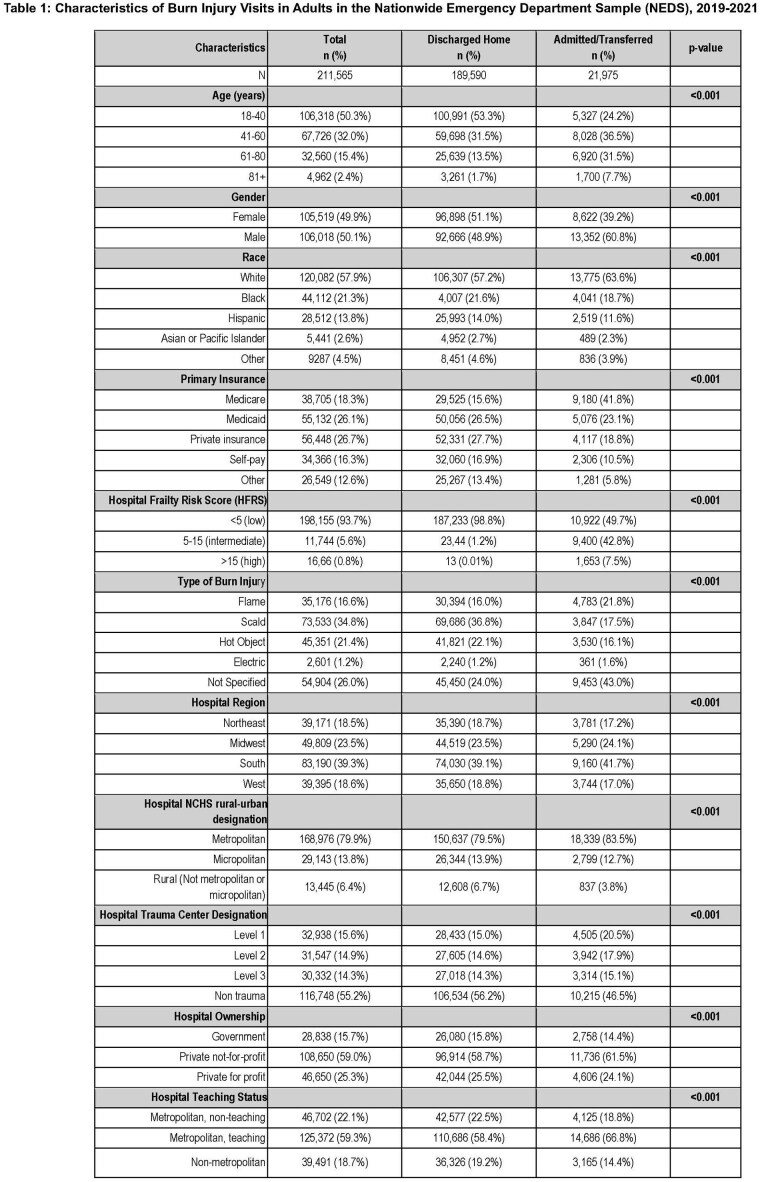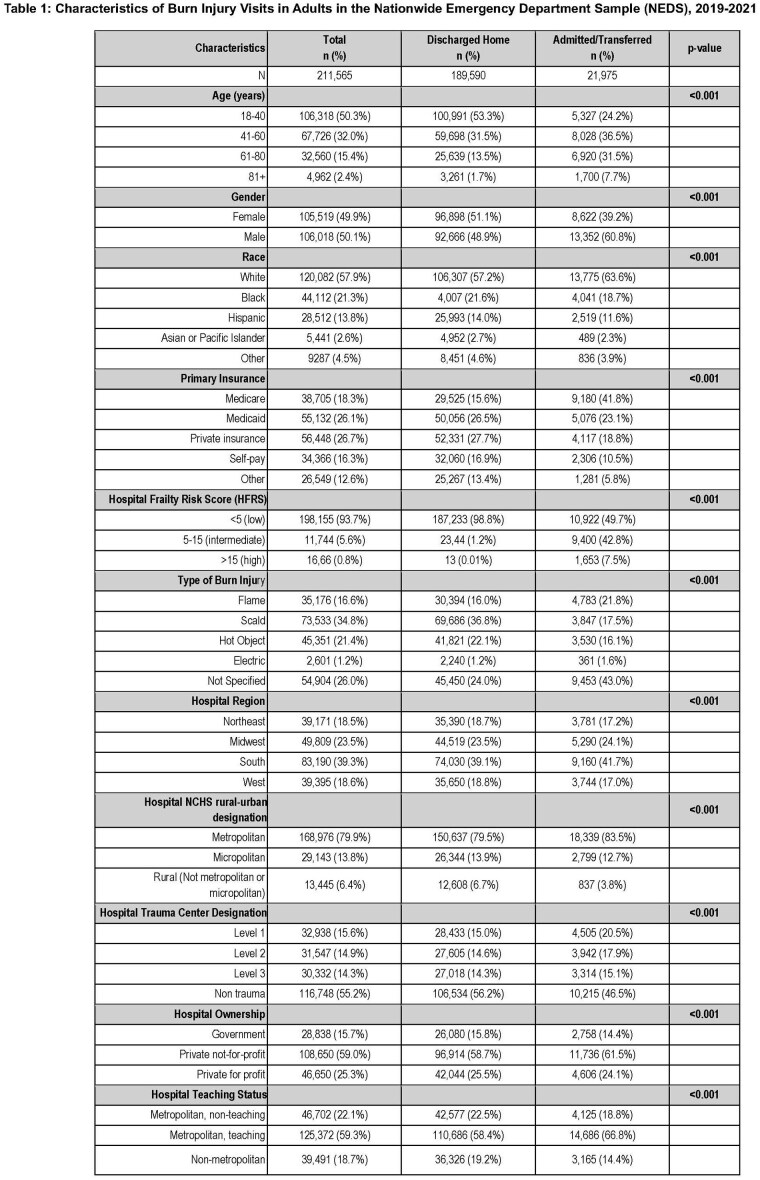# 604 Emergency Department Disposition Among Older Americans with Burn Injury

**DOI:** 10.1093/jbcr/iraf019.233

**Published:** 2025-04-01

**Authors:** Mary Hunter, Asha Chowanic, Zhaohui Fan, Naveen Sangji, Pasithorn Suwanabol, Mark Hemmila

**Affiliations:** University of Michigan Trauma Burn Center; University of Michigan School of Nursing; University of Michigan, Center for Healthcare Outcomes & Policy; University of Michigan, Division of Acute Care Surgery; University of Michigan, Division of Colorectal Surgery; University of Michigan, Division of Acute Care Surgery

## Abstract

**Introduction:**

Older adults (those greater than 65 years of age) represent 17% of the U.S. population and 10% of burn injured patients. Such patients are distinctly different than their younger counterparts given their higher levels of frailty, increased rates of comorbid conditions, and altered physiologic responses to injury. In this context, we sought to better understand the impact of burn injury among this growing and vulnerable population.

**Methods:**

We queried the Nationwide Emergency Department Sample (NEDS) for burn injury visits among all patients 18 years and older between 2019 and 2021. This data was weighted to allow for national estimates. Patients were categorized into age groups (18-40, 41-60, 61-80, 80+ years old). Frailty was quantified using the Hospital Frailty Risk Score (HFRS), which uses ICD-10 codes to stratify patients. Multivariable logistic regression analysis was used to assess the association of emergency department (ED) visit disposition and age, adjusting for gender, race, primary insurance, frailty, burn mechanism and hospital-level characteristics.

**Results:**

Between 2019 and 2021, there were 211,565 visits for adult burn injury. Of these, 90% (n=189,590) resulted in a discharge to home. Older adults had decreased odds of discharge home from the ED compared with younger adults (61-80 year-olds: OR 0.81, [95% CI: 0.76-0.87]; 81+ year-olds: OR 0.57 [95% CI: 0.51-0.64]). Furthermore, there was a strong correlation with frailer patients having lower odds of discharge to home from the ED (High HFRS: OR < 0.001 [95% CI: < 0.001-0.001]; Intermediate HFRS: OR 0.02 [95% CI: 0.021-0.024]).

**Conclusions:**

Older, frail adults have the highest association with non-home discharge following burn injury visits in the emergency department. Next steps in research can examine ways to optimize burn care for older adults and identify interventions that can facilitate a return to home after burn injury.

**Applicability of Research to Practice:**

This study identifies an important target for research in how burn injury affects older and frailer adults in the United States.

**Funding for the Study:**

N/A